# A Novel *ABCA12* Mutation in Two Families with Congenital Ichthyosis

**DOI:** 10.6064/2012/649090

**Published:** 2012-12-31

**Authors:** D. M. Walsh, S. H. Shah, M. A. Simpson, N. V. Morgan, S. Khaliq, R. C. Trembath, S. Q. Mehdi, E. R. Maher

**Affiliations:** ^1^Centre for Rare Diseases and Personalised Medicine, University of Birmingham, Edgbaston, Birmingham B15 2TT, UK; ^2^Centre for Human Genetics, Sindh Institute of Urology and Transplantation, Karachi 74200, Pakistan; ^3^Division of Genetics and Molecular Medicine, King's College London School of Medicine, Guy's Hospital, London, UK; ^4^University of Health Sciences, Lahore, Pakistan; ^5^West Midlands Regional Genetics Service, Birmingham Women's Hospital, Edgbaston, Birmingham B15 2TT, UK

## Abstract

Autosomal recessive congenital ichthyosis (ARCI) is a rare genetically heterogeneous disorder characterized by hyperkeratosis in addition to dry, scaly skin. There are six genes currently known to be associated with the disease. Exome sequencing data for two affected individuals with ichthyosis from two apparently unrelated consanguineous Pakistani families was analysed. Potential candidate mutations were analysed in additional family members to determine if the putative mutation segregated with disease status. A novel mutation (c.G4676T, p.Gly1559Val) in *ABCA12* occurred at a highly conserved residue, segregated with disease status in both families, and was not detected in 143 control chromosomes. Genotyping with microsatellite markers demonstrated a partial common haplotype in the two families, and a common founder mutation could not be excluded. Comparison to previously reported cases was consistent with the hypothesis that severe loss of function *ABCA12* mutations are associated with Harlequin Ichthyosis and missense mutations are preferentially associated with milder phenotypes. In addition to identifying a possible founder mutation, this paper illustrates how advances in genome sequencing technologies could be utilised to rapidly elucidate the molecular basis of inherited skin diseases which can be caused by mutations in multiple disease genes.

## 1. Introduction

Autosomal recessive congenital ichthyosis (ARCI) is a rare and genetically heterogeneous disorder characterized by hyperkeratosis in addition to dry, scaly skin [1]. It is a rare condition, with an estimated prevalence of approximately 1 in 200,000 births [2]. ARCI consists of three major subtypes, among which the range of clinical features and severity of disease vary [3]. The subtypes include Harlequin ichthyosis (HI), which is the most severe and devastating form of ichthyoses and is fatal in the majority of affected neonates [4, 5]. Lamellar ichthyosis (LI) and congenital ichthyosiform erythroderma (CIE) make up the further two subtypes and are not as severe as HI. The HI patients present with critical and significant clinical features at birth, including severe ectropion, eclabium, and flattened ears, in addition to large plate-like scales over the entire body [4, 6]. The patients who suffer from CIE on the other hand exhibit fine scales in addition to variable erythroderma, while LI patients show less severe erythroderma, but have thick, dark scales over the entire body [6, 7]. To date, multiple genes have been shown to be associated with ichthyosis, including *ALOXE3*, *ALOX12B*, *TGM1*, *CYP4F22*, *NIPAL4,* and *ABCA12* [3, 6]. Mutations in the *ABCA12* gene have been identified in all three subtypes of ARCI. The resulting defect in the ABCA12 protein is thought to play a causative role in the development of the disease by affecting lipid transport, which consequently causes abnormal development of the skin barrier [3, 8]. Here we report a novel missense mutation in exon 32 of *ABCA12* (p.Gly1559Val) that is associated with the development of congenital ichthyosis in two apparently unrelated families of Pakistani origin.

## 2. Materials and Methods

### 2.1. Patient Information

**Figure 1:**
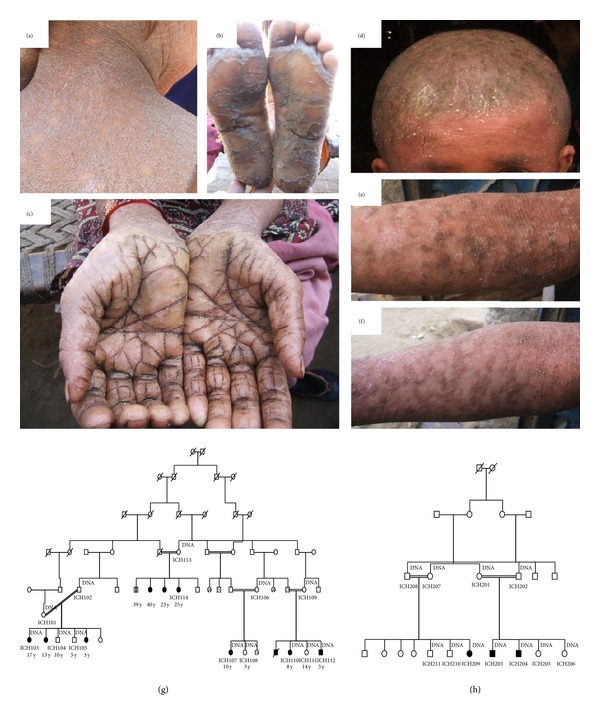
(a) Clinical features for several patients from family 1 with ichthyosis: proband from family 1 shows dry, cracked skin and hyperkeratosis on the bottom of the feet. (b) Clinical features for several patients from family 1 with ichthyosis: another member of family 1 exhibits fine, white scales in addition to erythroderma over entire body. (c) Clinical features for several patients from family 1 with ichthyosis: severe hyperlinear palms on an additional member of family 1. (d) Clinical features for several patients from family 2 with ichthyosis: the patient exhibits yellowish adherent scales and dry skin on the scalp. In addition, the patient displays fine white scales over the entire body and erythroderma. (e) and (f) Clinical features for several patients from family 2 with ichthyosis: hyperkeratosis can be observed on the feet, knees, and elbows (b and c). The patient and the other affected individuals in the family all have hypohidrosis and have severe heat intolerance. The affected patients in family 2 also have difficulty in breathing and allergic rhinitis. (g) Anonymous pedigree information for family 1: members included are labelled by ICH followed by a number. (h) Anonymous pedigree information for family 2.

Two consanguineous Asian families from the remote area of the Sindh province of Pakistan were ascertained, clinically assessed, and provided blood samples for DNA analysis. For both families, DNA was available for most of the affected individuals, at least one of their parents, and some unaffected individuals (total 8 affected and 17 unaffected family members (see Figures 1(g)-1(h)). The range of clinical symptoms varied among the affected individuals; however all presented with dry, scaly skin and hyperkeratosis to the elbows, knees, feet, and hands. 

### 2.2. Exome Sequencing

Exome sequencing was undertaken in two individuals (ICH107 and ICH204) according to the protocols used in the paper by Ostergaard et al. 2011 [9].

### 2.3. Microsatellite Markers

Microsatellite markers flanking *ABCA12 *were used to establish linkage within the two families. The following microsatellite markers were chosen: D2S2944, D2S2382, D2S164, and D2S434. PCR reactions were carried out and analysed.

### 2.4. Mutation Analysis of Candidate Genes

Primers were designed to flank either side of the coding exon harbouring the *ABCA12* mutation using ExonPrimer [10] and were then used to determine whether the mutation segregated in additional family members by conventional Sanger sequencing. 

## 3. Results

### 3.1. Clinical Features

Both families were known to be consanguineous from first cousin marriages (Figures 1(g)-1(h)). The affected individuals from family 1 presented with fine white scales over the entire body, in addition to dry skin and hyperkeratosis of the hands, feet, elbows, and knees (Figures 1(a)–1(c)). The hair of the patients seemed very dry, brittle, and thin. Deep pits were present in the toenails, and they seemed to be dystrophic. The individuals could not sweat and therefore were heat intolerant. The affected individuals in family 2 similarly showed fine white scales over the entire body in addition to rough, dry skin (Figures 1(d)–1(f)). All patients had generalized pruritis and exhibited erythematous and eczematous lesions and hypohidrosis and severe heat intolerance.

### 3.2. Analysis of Exome Sequencing Data

Whole exome sequencing data was obtained for the proband of each family. Filtering of the exome variant data was performed to identify homozygous previously unreported nonsynonymous changes. This reduced the number of candidate variants from the original 29,403 to 51 variants in family 1 and from 29,526 to 51 variants in family 2. 

**Figure 2:**
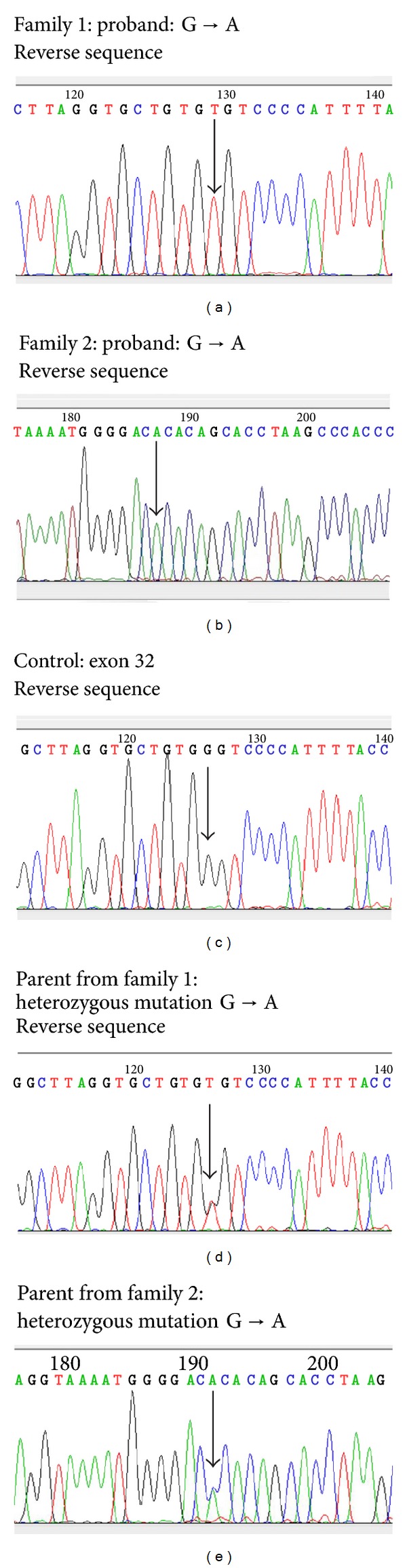
(a) Sequence analysis of ichthyosis patients: reverse sequence data showing a homozygous mutation present in the proband from family 1; the parents were both heterozygous carriers of this change. (b) Sequence analysis of ichthyosis patients: the sequence data for family 2 shows the heterozygous genotype of one of the parents. (c) Sequence analysis of ichthyosis patients: the mutation was not identified in any of the ethnically matched controls; all were found to be wildtype at this position.

There are 6 genes currently known to be associated with ichthyosis: *ALOXE3*, *ALOX12B*, *TGM1*, *CYP4F22*, *NIPAL4,* and *ABCA12*, and although thought to be unrelated, both the probands from family 1 and family 2 harboured an identical novel missense variant, c.G4676T, p.Gly1559Val in *ABCA12, *(see Figure 2). 

The exomic sequence flanking the putative *ABCA12 *mutation was amplified and sequenced from the additional 23 affected and unaffected family members. All affected individuals were homozygous for the c.G4676T mutation, all parents were heterozygous carriers, and unaffected siblings were either heterozygous carriers of the variant or homozygous wild-type (Figure 2). The mutation was not found in any of the 143 ethnically matched control chromosomes analysed. 

### 3.3. Genotype Analysis

In order to investigate whether the c.G4676T mutation was likely to represent a founder mutation from a common ancestor or was likely to have arisen independently, 4 affected individuals from the two families were genotyped at four polymorphic microsatellite loci flanking *ABCA12 *(D2S2944, D2S2382, D2S164, and D2S434). Identical genotypes were present in both families at *D2S2944,* the closest marker to *ABCA12*, but different alleles were detected at D2S2382 (which maps distal to *D2S2944*) (Table 1). This may be due to a recombination event which may have occurred between *ABCA12* and D2S2382, and, therefore, whilst the c.G4676T mutation might have occurred independently in the two families, a distant common ancestor cannot be excluded. 

## 4. Discussion

**Figure 3:**
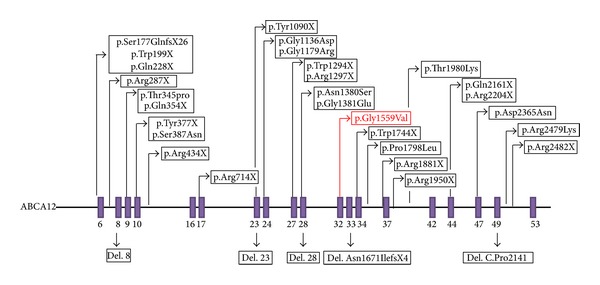
ABCA12 gene showing the locations of some mutations already identified and location of novel missense change. The exons in the gene are shown in purple. Missense changes can be found above the gene, while deletions can be found underneath the gene. The novel missense change identified in the two families is marked in red at exon 32.

**Figure 4:**
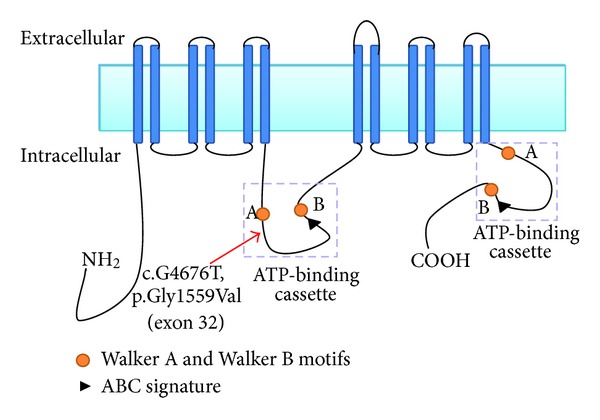
Schematic representation of the ABCA12 protein and predicted location of mutation. The mutation will exert its effect on the Walker A motif of the 1st ATP-binding cassette; an area within the nucleotide-binding domain.

Despite the genetic heterogeneity of autosomal recessive congenital ichthyosis [3, 6], exome resequencing and prioritisation of homozygous novel variants (in view of the consanguinity present in both families) enabled us to rapidly identify a candidate *ABCA12* novel missense mutation. We then confirmed likely pathogenicity by segregation analysis in families and excluding the variant in ethnically matched controls. The *ABCA12* gene encodes the ATP-binding cassette subfamily A member 12 protein that belongs to a family of proteins implicated in the regulation of the transport of various molecules across cell membranes [11–13]. It is a keratinocyte transmembrane lipid transporter protein which has been shown to be expressed in various cells and tissues, including skin cells, placenta, lung, stomach, and liver [13, 14]. *ABCA12 *appears to be essential for the normal development of the epidermis, and mutations in the gene may cause defective lipid transport which consequentially leads to abnormalities in the formation of the epidermal lipid barrier [11]. Although mutations in *ABCA12 *are known to be associated with Ichthyosis, and many different mutations within this gene have been documented (Figure 3), the missense mutation detected in the two families we investigated had not previously been described. The segregation of the mutation within two large families, absence of the mutation in South Asian controls, and the observation that the substitution occurs at a residue that is evolutionarily conserved in all mammals, chicken, and zebrafish are all consistent with a pathogenic mutation. The p.Gly1559Val substitution maps an intracellular loop that contains the motif of the ATP-binding cassette (see Figure 4). 

Our findings of a novel *ABCA12* missense mutation further contribute to the mutational diversity of *ABCA12*-related ichthyosis and will facilitate structure-function and genotype-phenotype correlations. The finding of a CIE phenotype associated with a p.Gly1559Val missense substitution is consistent with previously reported genotype-phenotype studies (see Supplementary Table available online at http://dx.doi.org/10.6064/2012/649090 and Table 2) [15]. Thus in patients with an HI phenotype, truncating mutations and deletions predominate whereas missense mutations are more common in less severe cases (CIE and LI) [16]. All patients with HI harbour at least one deletion or a truncation mutation [15]. Thus it is suggested that the p.Gly1559Val substitution, located in the first ATP-binding cassette, produces a partial loss of ABCA12 function.

This paper illustrates how advances in second generation sequencing can greatly facilitate the molecular diagnosis of inherited skin diseases for which a single phenotype can be caused by multiple genes. Though the current standard molecular diagnostic approach is to sequentially sequence the individual genes, second generation sequencing enables all the potentially relevant genes to be captured and sequenced simultaneously–either by disease-specific panels of genes or by exome/partial exome strategies which could provide a standard approach to be utilised for many different rare diseases.

## Supplementary Material

Table listing associated phenotypes of known ichthyosis *ABCA12* mutations [15].

## Figures and Tables

**Table 1:** Microsatellite data collected for the four affected individuals with ichthyosis: the affected individuals were all found to be homozygous for the c.G4676T, p.Gly1559Val mutation. It should be noted that the individual ICH103 showed a slight shift in the haplotype when compared to family 2. This could be due to the fact that the variants developed independently in both families. However, it may also mean that the variants were inherited by a common ancestor and have undergone a recombination event between *ABCA12* and DSS164.

Marker	Physical Location (Mb)	ICH103	ICH203	ICH204	ICH209
D2S2944	21.4	107	107	107	107
*ABCA12 *	21.5				
D2S2382	21.6	329	306	306	306
D2S164	21.7	274	—	274	274

**Table 2:** A summary of the types of mutations identified (from the literature review) for each subtype of ARCI (autosomal recessive congenital ichthyosis) and the corresponding effects on the ABCA12 protein. This table shows that the most deleterious changes often result in the HI phenotype, while the mutations which have a less severe effect on the protein often lead to the CIE and LI phenotypes.

Number of patients	Phenotype	Mutations
42	HI	4.8% frameshift insertions
		7.1% exonic deletions
		9.5% in-frame deletions
		*9.5% missense*
		11.9% splice site
		16.7% frameshift deletions
		40.5% nonsense

11	CIE	*72.7% missense*
		27.3% nonsense

5	LI	*100% missense*

HI: Harlequin ichthyosis.

CIE: congenital ichthyosiform erythroderma.

LI: lamellar ichthyosis.
