# Supplementary material for: A Novel ABCA12 Mutation in Two Families with Congenital Ichthyosis
Source: Scientifica (Cairo). 2012 Dec 31;2012:649090. doi: 10.6064/2012/649090 (PMC3820470; doi:10.6064/2012/649090)
Supplement: Supplementary file 1 — Table listing associated phenotypes of known ichthyosis ABCA12 mutations [15]. [file 649090.f1.pdf]

Supplementary Information

Supplementary Table 1. A list of the genotypes and associated phenotypes of all known ichthyosis *ABCA12* mutations [15]. Also included is information regarding the domain of the protein affected by the mutation, and the consequence to the protein [6, 15].

Exon	Genotype	Phenotype	Protein Domain	Effect on Protein
2	p.I38HisfsX64, c.107_108insT	HI	N-terminal	Truncation
6	p.Ser177GlnfsX, c.529delT	HI	N-terminal	Truncation
6	p.Trp199XX, c.G596A	HI	N-terminal	Truncation
6	p.Gln228X, c.C682T	HI	N-terminal	Truncation
7	p.Arg287X, c.C859T	HI	N-terminal	Truncation
8	Exon 8 Deletion	HI	N-terminal	Non-truncation
9	p.Thr345Pro, c.A1033C	CIE	N-terminal	Non-truncation
9	p.Gln354X, c.C1060T	HI	N-terminal	Truncation
10	p.Tyr377X, c.C1131G	HI	N-terminal	Truncation
10	p.Ser387Asn, c.G1160A	HI	N-terminal	Non-truncation
12	p.Arg434X, c.C1300T	HI	N-terminal	Truncation
15	p.Trp601X, c.G1803A	HI	N-terminal	Truncation
16	p.Lys674ArgfsX49, c.2021_2022del2	HI	N-terminal	Truncation
16	p.Ile676PhefsX13, c.2025delG	HI	N-terminal	Truncation

17	p.Arg714X, c.C2140T	HI	N-terminal	Truncation
17	p.Leu758PhefsX4, c.2274_2275insT	HI	N-terminal	Truncation
23	p.Tyr1090X, c.3270delT	HI	Transmembrane domains	Truncation
23	p.Tyr1090X, c.T3270G	HI	Transmembrane domains	Truncation
23	Exon 23 Deletion	HI	Transmembrane domains	Truncation
Intron 23	Abnormal splicing, c.A3295-2G	HI	Transmembrane domains	Truncation
24	p.Gly1136Asp, c.G3407A	CIE	Transmembrane domains	Non-truncation
24	p.Gly1179Arg, c.G3535A	HI	Transmembrane domains	Non-truncation
25	p.Arg1225X, c.C3673T	HI	Transmembrane domains	Truncation
26	p.Trp1235Ser, c.G3704C	CIE	Transmembrane domains	Non-truncation
26	p.Ser1249X, c.C3746A	HI	Transmembrane domains	Truncation
Intron 26	Unknown, c.3829+1G>A	HI	Unknown	Unknown
27	p.Trp1294X, c.G3882A	HI	Transmembrane domains	Truncation
27	p.Arg1297X, c.G3891A	HI	Transmembrane domains	Truncation
28	p.Asn1380Ser, c.A4139G	LI	1st ATP-binding cassette	Non-truncation
28	p.Gly1381Glu, c.G4142A	LI	1st ATP-binding cassette	Non-truncation
28	p.Thr1387del, c.4158_4160del3	HI	1st ATP-binding cassette	Non-truncation
28	Exon deletion (exon 28- 53)	HI	1st ATP-binding cassette onwards	Truncation
29	p.Gly1421GlufsX39, c.4262delG	HI	1st ATP-binding cassette	Truncation

30	p.Ile1494Thr, c.T4481C	CIE	1st ATP-binding cassette	Non-truncation
30	p.Arg1514His, c.G4541A	CIE, LI	1st ATP-binding cassette	Non-truncation
31	p.Glu1539Lys, c.G4615A	LI	1st ATP-binding cassette	Non-truncation
**32**	**p.Gly1559Val, c.G4676T**	**CIE**	**1st ATP-binding cassette**	**Non-truncation**
32	p.Gly1651Ser, c.G4951A	LI	1st ATP-binding cassette	Non-truncation
33	p.Gln1669X, c.C5005T	CIE	Unknown	Truncation
33	p.Asn1671IlefsX4, c.5012delA	HI	Unknown	Truncation
33	c.5125_5128del4	HI	Unknown	Truncation
34	p.Trp1744X, c.G5231A	HI	Transmembrane domains	Truncation
Intron 34	Unknown, c.5381+3_5381+4del2	HI	Unknown	Unknown
35	p.Pro1798Leu, c.C5393T	CIE	Unknown	Non-truncation
37	p.Arg1881X, c.C5641T	HI	Unknown	Truncation
37	c.G5690T	HI	Unknown	Unknown
39	p.Arg1950X, c.C5848T	HI, CIE	Unknown	Truncation
40	p.Thr1980Lys, c.C5939A	CIE	Unknown	Non-truncation
42	p.Ala2054AspfsX10, c.6160_6161del2	HI	Transmembrane domains	Truncation
42	c.G6234T	HI	Unknown	Unknown
44	p.Pro2141del, c.6443_6445del3	HI	Transmembrane domains	Non-truncation
44	p.Gln2161X, c.C6481T	HI	Transmembrane domains	Truncation

44	p.Arg2204X, c.C6610T	HI	Transmembrane domains	Truncation
47	p.Asp2365Asn, c.G7093A	HI	2nd ATP-binding cassette	Non-truncation
49	p.Val2442SerfsX28, c.7322delC	HI	2nd ATP-binding cassette	Truncation
50	p.Arg2479Lys, c.G7436A	HI	2nd ATP-binding cassette	Truncation
51	p.Arg2482X, c.T7444C	HI, CIE	2nd ATP-binding cassette	Truncation
